# Acknowledgement to reviewers

**DOI:** 10.1530/RAF-7-A1

**Published:** 2026-02-06

The editorial board wishes to thank the following individuals who have helped to review manuscripts for *Reproduction & Fertility* during 2025. Their assistance is gratefully acknowledged.

F Acuña

F Ahmed Khan

S Aikawa

R Aitken

N Akin

A Al Ahmar

H Al Ahwany

S Altmae

E Alves

F Amant

F Amargant

O Ammar

C Amorim

J Andonissamy

P Arakaki

P Archary

B Asimakopoulos

C Aurich

M Balbach

D Bateson

M Bellingham

M Binelli

J Bloom

A Boediono

K E Boers

E Bolcun-Filas

M Bonato

A Boyd

T Bracewell-Milnes

Z Breton

I Bustamante-Filho

M Canis

J Carrillo

Q Chen

X Chen

H Chen

M-J Chen

Y Cheong

E Cheraghi

S Cho

J Chow

R K W Choy

J Cleland

A Converse

A E Crosier

S Daryabari

J Davis

A de Mestre

D de Ziegler

T DeFalco

E Delgouffe

H Diao

F Diaz

P Dini

R Domingues

D Duffy

W C Duncan

A Ealy

I A Edagha

G Eleje

A El-Mazny

N Ennis

R Fathi

S Freger

R Freiman

A Fukui

S Gacem

S Gamal El Din

S Garwin

E Gastal

S Gellatly

T Gerrits

N Ghanami Gashti

S Ghersevich

M Gholiof

M Giassetti

J Goo

A Gram

E Güell

D Guerreiro

I Guisado-Cuadrado

G Gullo

K Halloran

A Harrer

A Hawdon

X He

K M Hellman

A Hewett

F Hollinshead

T Hopkins

Y Hou

B J Houston

B Huang

C Hughes

M Ibrahim

J Ito

H Iwata

V Jagannathan

N Jahangiri

D Ježek

P N Jorge-Neto

J Kang

S Karunakaran

C Kerestes

M A Khan

S C Khaw

D Kimelman

A Kirkland

M J Kmiecik

K Knapczyk-Stwora

P Koninckx

R Kordus

L Kullik

B Kumar

A Kumar

S Kumar

R Kuon

N Kuscu

C Kyriacou

D Lamb

M Laronda

B Lawrence

N Q K Le

S Le Gac

MS Lee

M Leonardi

B Levandowski

M Li

H W R Li

Y Liang

G Liperis

H Liu

V Lodde

D Logsdon

F Lone

A Luciano

Z Mahmoud

M Majidi Zolbin

E Malama

C Martins

S Martins da Silva

M Masjedi

C Massarotti

C Matás Parra

S McAllister

S McMenamin

M Meel

S Mehdinejadiani

G Merges

M Miglino

J Miles

J Mitsui

T Mohamed

T Mohanty

C Monaco

S Moody

C Mulder

P Nag

J Nagashima

S Nagy

M H Nasr-Esfahani

L Navarro-Sánchez

A Nelson

M Neves

K Ng

P R Nunes

J Odendaal

Y Odey

S Ombelet

J Omole-Mathew

M O'Reilly

C Owens

S Pandey

A Paping

J A Pasternak

N Paul

K Pavani

R Payan-Carreira

S Penir

D Pepin

F Petitprez

E Pinart

E Pintus

CS Pizzutto

E Przygrodzka

M Qasemi

A Quinn

M Quinn

M Rae

MA Rahman

M Ralapanawe

S Relovska

E A Rhon-Calderon

M Rimmer

J Roca

R Rosario

P Ruane

I S Saadeldin

M Sagharichi

N Sah

P Saha

S R Salian

W Samson

R Sapiro

P T Saunders

A Seitz

H Seo

M Serdarogullari

G Serour

C Shadowen

D Sharkey

G Sharma

S Sharma

N Sheibak

M Shen

M Shokoohi

S Silber

A R Silva

M Silvestre

M Simopoulou

S Simsek

M K Sinha

P Sirayapiwat

M Skrzypski

H Smith

K Smits

D Soffa

A Soler

M A Spadella

A Stefansdottir

D Swain

T H Swartz

A Swegen

G Szabo

F Tarnonsky

M Tavalaee

E Thabet

D Thaldar

I Thanaboonyawat

H Tinning

F Topbas Selcuki

J Torres Carreira

N Trigg

J Uloho

S B Utami

C C Vaduva

G van der Horst

L Vansandt

J Veltman

F Vitale

L Vrooman

K Wallace

A Watkins

L Wehrli

R West

J Wilkinson

E Williams

S Williams

B Wiweko

A Woolner

H-M Wu

M-H Wu

W Xin

B Xu

H Xu

W Xu

S Yeatman

E J Yu

M Yuan

A Ziecik

